# Bioprinted skin: from lab bench to clinic, what matters now and what’s next

**DOI:** 10.3389/fbioe.2026.1782511

**Published:** 2026-06-26

**Authors:** Diala Haykal, Frédéric Flament, Pascale Mora, Bambou Tan, Caroline Sirichandra

**Affiliations:** 1 Centre Laser Palaiseau, Private Practice, Palaiseau, France; 2 L’Oreal Research and Innovation, Clichy, France; 3 Episkin, Lyon, France

**Keywords:** animal-free testing, artificial intelligence, biofabrication, bioprinting, imaging innovation, plastic surgery, regenerative medicine, tissue engineering

## Abstract

Bioprinted skin has emerged as a disruptive technology at the intersection of tissue engineering and plastic surgery. Through controlled deposition of bioinks containing living cells and biomaterials, it is now possible to create human skin constructs with unprecedented physiological fidelity. These constructs are already transforming the evaluation of drugs and cosmetics by providing reproducible, human-relevant alternatives to animal testing. In reconstructive and aesthetic surgery, bioprinted skin holds the promise of patient-specific grafts that integrate seamlessly, accelerate healing, and minimize scarring and pigmentation mismatch. This article explores the clinical, technical, and translational dimensions of bioprinted skin, with particular emphasis on fabrication strategies, bioink design, and current limitations. It further highlights the role of imaging, artificial intelligence, and advanced biomaterials in accelerating clinical translation while critically discussing the challenges that still prevent widespread implementation.

## Introduction

Replicating the structural, cellular, and functional complexity of human skin has long represented a frontier in biomedical engineering. Native skin is a multilayered organ composed of the epidermis, dermis, and hypodermis, integrating multiple cell types, extracellular matrix components, vascular networks, and appendages. Reproducing this level of organization *in vitro* requires not only cellular diversity but also precise spatial arrangement and biochemical signaling.

Recent advances in three-dimensional (3D) bioprinting have brought this goal closer to reality by enabling the controlled, layer-by-layer deposition of living cells, biomaterials, and signaling molecules into constructs that reproduce epidermal and dermal microarchitecture. Once confined to experimental research, bioprinting has rapidly evolved into a translational platform with growing relevance for dermatology, plastic surgery, and regenerative medicine.

Beyond reconstructive potential, bioprinted skin offers unparalleled opportunities to model disease, test therapeutic compounds, and replace traditional animal experimentation. The integration of biofabrication, artificial intelligence, and advanced materials science is accelerating progress toward clinically functional, patient-specific grafts capable of seamless tissue integration and regeneration (Ahn et al., 2023).

## From experimental fabrication to clinical utility

Restoring the structural and functional integrity of the skin has long been a central challenge in reconstructive and plastic surgery. Traditional autologous grafts, although effective, are limited by donor-site morbidity, restricted surface availability, and variability in pigmentation and texture. Allogeneic grafts and synthetic matrices, on the other hand, are frequently associated with immune rejection, suboptimal integration, and limited long-term durability (Haykal et al., 2026).

Three-dimensional bioprinting introduces a fundamentally different approach by enabling the fabrication of skin constructs through controlled deposition of bioinks, which are hydrogel-based systems containing living cells, extracellular matrix components, and bioactive molecules. These bioinks are engineered to exhibit specific rheological properties, such as shear-thinning behavior, allowing extrusion while preserving cell viability (Derman et al., 2025; Lai et al., 2024).

Different bioprinting modalities, including extrusion-based, inkjet, and laser-assisted techniques, enable varying degrees of precision and cell density. Extrusion-based systems are most commonly used for skin fabrication due to their compatibility with viscous, cell-rich bioinks, whereas laser-assisted systems offer higher resolution at the expense of complexity.

Through these approaches, bioprinted grafts can be tailored to match defect geometry, thickness, and even pigmentation. Importantly, constructs are typically fabricated in a hierarchical manner, with a dermal layer containing fibroblasts embedded in collagen-rich matrices, followed by an epidermal layer populated with keratinocytes. This architectural organization is critical for restoring barrier function and mechanical integrity (Albanna et al., 2019).

## How 3D bioprinting fabricates skin grafts: technical principles

### Overview of the bioprinting workflow

The fabrication of a bioprinted skin graft follows a multi-stage pipeline. First, patient-derived cells (typically keratinocytes and fibroblasts, and increasingly melanocytes, endothelial cells, and pericytes) are harvested via biopsy and expanded *ex vivo* under GMP-compliant cell culture conditions. These cells are then suspended in bioinks, hydrogel-based carriers engineered to support viability during printing and to promote subsequent matrix remodeling (Weng et al., 2021).

**FIGURE 1:**
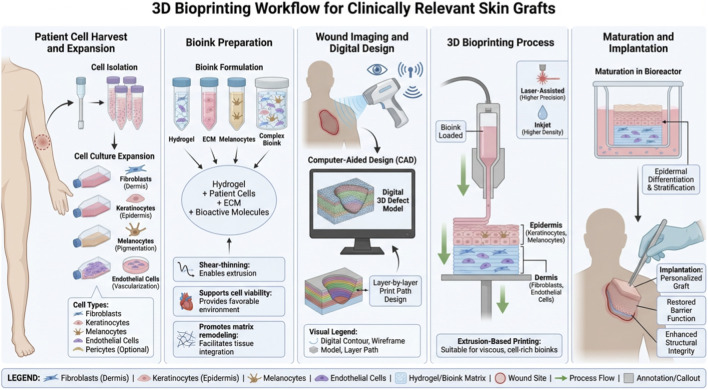
3D bioprinting workflow for clinically relevant skin grafts.

A digital 3D model of the target wound or defect is obtained through optical or ultrasound scanning. Computer-assisted design software translates this topography into a layer-by-layer print path. The bioprinter then deposits the bioink according to this path, building the construct from the dermis upward. The resulting structure is matured in a bioreactor or at an air-liquid interface to promote epidermal differentiation before implantation or use (Dandoulakis, 2026) (Figure 1).

### Bioprinting modalities

Extrusion-based bioprinting is the most widely adopted platform for skin applications. Bioink is extruded through a nozzle under pneumatic or mechanical pressure in a continuous filament. It is compatible with high-viscosity, cell-rich hydrogels and enables fabrication of large-area constructs. However, shear forces during extrusion can reduce cell viability, and spatial resolution is lower than laser-based methods (Baltazar et al., 2020).

Inkjet Drop-On-Demand (DOD) bioprinting ejects discrete droplets of bioink onto the substrate using piezoelectric, thermal, or electrostatic actuation. It offers higher resolution and non-contact deposition, reducing contamination risk. The main limitation is the requirement for low-viscosity bioinks, which restricts cell density and structural stability. The small aperture (10–150 µm) is also prone to clogging.

**FIGURE 2:**
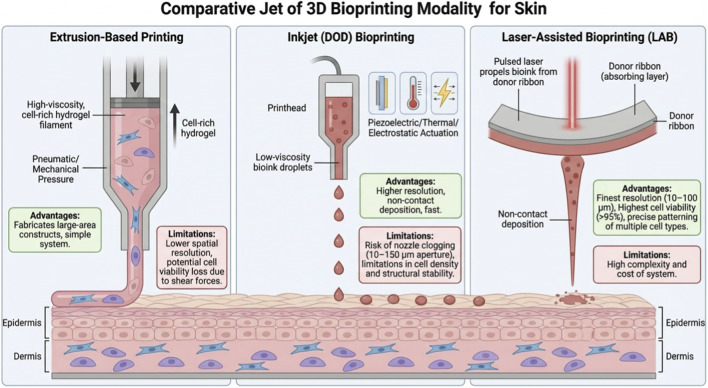
Comparative jet of 3D bioprinting modality for skin.

Laser-assisted bioprinting (LAB) uses a pulsed laser to propel bioink from a donor ribbon onto the substrate without direct contact, achieving the finest resolution (10–100 µm) and highest cell viability (>95%). LAB is particularly suited to precise patterning of multiple cell types, though system complexity and cost limit its widespread adoption (Gudapati et al., 2016; Lee et al., 2018) (Figure 2) (Table 1).

**TABLE 1:** Comparative overview of 3D bioprinting modalities for skin applications (Baltazar et al., 2020; Gudapati et al., 2016; Lee et al., 2018).

Parameter	Extrusion-based	Inkjet (DOD)	Laser-assisted (LAB)
Spatial resolution	Low–medium	Medium	High
Compatible bioink viscosity	High	Low	Low–medium
Cell viability post-print	Moderate	Good	Excellent
Scalability for large constructs	High	Medium	Low
System cost and complexity	Low	Medium	High
Primary limitation	Shear stress on cells	Nozzle clogging	Cost and complexity

### Bioink design and rheological requirements

Effective bioink formulation requires balancing two competing demands: the material must be fluid enough to pass through a nozzle or droplet system during printing, yet stable enough to hold its shape immediately after deposition. This behavior is governed by shear-thinning rheology, under the mechanical stress of extrusion, the bioink’s viscosity drops to allow flow, then rapidly recovers once the shearing force is removed, preserving the printed architecture (Ashammakhi et al., 2019).

The choice of base material determines the scaffold’s biological and mechanical character. Collagen types I and III remain the most physiologically faithful options, closely mimicking the native dermal ECM and providing an adhesive substrate for fibroblast attachment and matrix remodeling. Gelatin methacrylate (GelMA) has emerged as a highly versatile alternative, offering tunable stiffness through UV photocrosslinking and strong biocompatibility across a range of cell types. Alginate crosslinks rapidly in the presence of calcium ions, making it easy to process, though its lack of native cell adhesion motifs typically requires chemical functionalization before it can support meaningful cell-matrix interactions. Beyond these established materials, fibrin, hyaluronic acid, and decellularized extracellular matrix (dECM) each bring distinct advantages, particularly dECM, which preserves the native biochemical complexity of the source tissue and provides an unmatched biological microenvironment for embedded cells (Shen et al., 2020).

Beyond rheological and mechanical considerations, the immunological compatibility of bioinks represents a critical determinant of successful clinical translation. Commonly used biomaterials, including collagen, GelMA, alginate, fibrin, and dECM, may actively modulate both innate and adaptive immune responses after implantation. Residual antigens or damage-associated molecular patterns (DAMPs) within dECM-derived bioinks can influence macrophage activation and downstream inflammatory signaling. Likewise, the choice of crosslinking strategy, whether UV-mediated, ionic, enzymatic, or thermal, affects degradation kinetics, inflammatory responses, and cellular compatibility through the generation of distinct degradation by-products. Increasing evidence also demonstrates that scaffold stiffness, matrix architecture, and degradation profiles can regulate macrophage polarization, fibroinflammatory remodeling, and T-cell recruitment, including regulatory T-cell responses. Importantly, even autologous cell-laden constructs may elicit innate immune activation driven primarily by the biomaterial scaffold itself rather than the cellular component. Consequently, future bioink development must integrate immunomodulatory considerations alongside mechanical fidelity and printability to improve graft integration, long-term stability, and functional tissue regeneration.

Once deposited, the bioink must be permanently stabilized through crosslinking. The mechanism chosen, whether UV photopolymerization, enzymatic crosslinking, ionic gelation, or thermal transition, profoundly influences both the mechanical durability of the construct and the survival of encapsulated cells. Increasingly, dual-crosslinking approaches that combine two complementary mechanisms are favored: rapid ionic or thermal pre-gelation arrests the structure immediately after deposition, while a secondary covalent crosslinking step builds the long-term mechanical integrity required for handling and implantation (Ouyang, 2022; Unagolla and Jayasuriya, 2020).

### Hierarchical skin architecture and cell layering

Fabricating a functional skin graft requires recapitulating the bilayered (or trilayered) architecture of native skin. In the standard approach, a dermal layer is first printed using a bioink containing fibroblasts embedded in a collagen-rich or GelMA matrix. This layer supports ECM synthesis, mechanical support, and vascular network formation. A second epidermal layer containing keratinocytes is then deposited atop the dermal compartment and induced to differentiate at an air-liquid interface, forming a stratified, cornified epithelium with barrier function (Kado Abdalkader et al., 2025).

Advanced constructs incorporate additional cell types: melanocytes for pigmentation matching, endothelial cells and pericytes for pre-vascularization, and stem cells for enhanced regenerative capacity. Patterned deposition using specialized bioprinting allows spatial control over cell distribution, for instance, placing melanocytes at the dermal-epidermal junction to replicate physiological pigmentation.

### Advantages of 3D bioprinting for skin graft fabrication

Compared with conventional split-thickness or full-thickness autografts, 3D bioprinted skin offers several structural and clinical advantages (Baltazar et al., 2023; Olejnik et al., 2022):

Customization to wound geometry: Digital wound scanning and CAD enable the fabrication of grafts precisely matched to defect shape, depth, and topography, eliminating the mismatch encountered with sheet grafts.

Patient-specificity: The use of autologous cells eliminates immune rejection and pigmentation mismatch. Constructs can be tailored to replicate the patient’s skin color, thickness, and mechanical properties, which is particularly relevant for facial and hand reconstruction.

Speed of production: Bioprinting can produce 100 cm^2^ of bilayered skin equivalent in under 35 min, a significant advantage over traditional 3-week culture timelines for comparable constructs.

Scalability: Automated printing platforms allow reproducible, large-area graft production under GMP conditions, addressing the scalability limitations of manual tissue engineering methods.

Functional integration of biologics: Growth factors, cytokines, exosomes, and other bioactive molecules can be embedded within the bioink and released in a spatiotemporally controlled manner, actively modulating the wound healing microenvironment.

### Current technical limitations

Despite these advances, critical technical challenges remain unresolved: (Hann et al., 2019; Chae et al., 2023; Fischer et al., 2026; Motter et al., 2023; Zhang et al., 2021):

Vascularization: The most significant barrier to clinical translation is the absence of a functional vascular network within the printed construct. Without perfused capillaries, the maximum nutrient diffusion distance that can sustain cell viability is approximately 100–200 μm, limiting graft thickness. Although pre-vascularization strategies embedding endothelial cells or sacrificial vascular channels have been explored, achieving rapid inosculation (anastomosis with host vessels within the 72-h critical window) remains elusive. Existing approaches typically achieve vascular integration only within 4 weeks of implantation in murine models.

Absence of skin appendages: Current bioprinted constructs universally lack functional hair follicles, sweat glands, sebaceous glands, and sensory nerve endings. These structures are critical for thermoregulation, sensation, and normal skin physiology. Their inclusion requires engineering organoid-like structures with precise 3D microenvironmental cues, which has not yet been achieved reproducibly.

Mechanical mismatch: The elastic and tensile properties of hydrogel-based bioprinted skin constructs do not fully replicate native skin, particularly for high-movement areas such as joints or the dorsum of the hand. Over time, differential mechanical stresses can lead to graft failure or aberrant remodeling.

Pigmentation fidelity: While melanocytes can be incorporated, achieving uniform and stable pigmentation that matches surrounding skin across different phototypes remains technically demanding, particularly in patients with darker skin tones.

Scalability and cost: High-quality bioprinters, GMP-grade cell culture, and bioink formulation remain expensive. Regulatory compliance demands extensive batch testing for viability, sterility, and mechanical integrity, further increasing production costs.

## A paradigm shift in plastic and reconstructive surgery

The first clinical trials using bioprinted autologous skin for burn coverage have demonstrated promising outcomes, with improved epithelialization, improved vascular integration, reduced contracture formation, and favorable graft take rates. Graft take rate is a classical surgical parameter describing the percentage of graft survival and successful integration with the wound bed following transplantation. One of the major innovations lies in the incorporation of pre-vascularized microarchitectures, such as endothelial cell-lined channels, which facilitate rapid inosculation with host vasculature and improve graft survival.

Unlike conventional sheet grafts, bioprinted constructs allow for spatial control over cellular distribution and matrix composition. This accelerates graft survival and reduces necrosis, a recurrent challenge in large or deep wounds (Zhang et al., 2025; Zhang et al., 2022).

In reconstructive surgery, the capacity to design grafts with regional variations in dermal thickness and pigmentation opens new possibilities for delicate zones such as the face, neck, and hands. Surgeons could soon print customized skin patches intraoperatively using patient-derived fibroblasts and keratinocytes expanded *ex vivo*. The combination of autologous cells and biomimetic matrices may not only restore coverage but also improve sensory recovery and reduce hypertrophic scarring, particularly when combined with adjunctive laser or energy-based therapies (Chen et al., 2021).

## Reducing animal testing and enhancing predictive science

Beyond its clinical applications, bioprinted skin represents a major ethical and scientific advancement by providing a reliable human-based alternative to animal models. Traditional models often fail to replicate human-specific features such as barrier function, pigmentation, and immune signaling pathways.

The European Union’s full ban on cosmetic animal testing, the OECD’s (Organisation for Economic Co-operation and Development) endorsement of reconstructed human epidermis models, and the U.S. FDA’s Modernization Act 2.0, which allows non-animal methods for drug development, are all driving a profound regulatory shift toward human-relevant models (Han, 2023; Alépée et al., 2019).

Bioprinted skin constructs are capable of reproducing key physiological processes, including keratinocyte differentiation, extracellular matrix deposition, and inflammatory responses. Advanced models incorporating melanocytes, immune cells, and endothelial components allow the study of complex conditions such as photoaging, pigmentation disorders, and impaired wound healing.

Moreover, the use of standardized biofabrication protocols improves reproducibility and enables quantitative assessment of toxicity, irritation, and sensitization. This shift toward biologically relevant systems is aligned with evolving regulatory frameworks and contributes to reducing animal experimentation while enhancing translational predictivity.

## Bioprinted skin as a model for dermatologic disease and therapy development

Beyond safety testing, bioprinted skin provides an unprecedented opportunity to model human dermatologic diseases with physiological accuracy. By incorporating genetically modified keratinocytes, fibroblasts, melanocytes, or immune cells, researchers can recreate pathological conditions such as psoriasis, atopic dermatitis, vitiligo, and photoaging within controlled *in vitro* environments. Recent innovations in bioprinting have enabled the creation of advanced bilayer scaffolds that precisely mimic natural skin architecture, significantly accelerating the development of fully differentiated skin models with a neosynthesized extracellular matrix for tailored tissue engineering (Girard et al., 2024).

Furthermore, specialized patterned bioprinting techniques have been utilized to construct heterogeneous reconstructed epidermal models, which are crucial for studying complex dermatological conditions such as atopic dermatitis or ichthyosis vulgaris by controlling the spatial deposition of different keratinocyte populations (Madiedo-Podvrsan et al., 2021).

These cells can also be genetically modified to express or suppress key genes, enabling the recreation of disease-specific phenotypes and the evaluation of targeted molecular therapies. This approach enables the study of inflammatory signaling, matrix remodeling, and pigmentary regulation under near-physiological conditions, while also facilitating the development and preclinical validation of gene- or pathway-targeted therapies. Such disease-relevant constructs not only accelerate drug discovery but also advance personalized medicine by predicting individual therapeutic responses before clinical application (Peng et al., 2025).

## A platform for innovation in wound management

The integration of bioprinted skin into wound care represents a major opportunity for innovation. Chronic wounds, including diabetic ulcers and radiation-induced injuries, are characterized by impaired vascularization and persistent inflammation, which limit healing.

Bioprinted constructs enriched with angiogenic factors, stem cells, or bioactive molecules can actively modulate the wound microenvironment. These “bioactive grafts” go beyond passive coverage by delivering controlled biochemical signals that promote angiogenesis, collagen deposition, and tissue remodeling (Bo et al., 2024).

The combination of bioprinting with imaging and computational tools further enables the personalization of graft composition based on patient-specific parameters, such as wound depth, metabolic status, and inflammatory profile (Tabriz and Douroumis, 2022; Biondo et al., 2024; Antezana et al., 2022).

## From prototypes to scalable manufacturing

To translate laboratory prototypes into clinical-grade grafts, bioprinting requires a robust framework for standardization, quality control, and reproducibility. The transition from research-scale printing to regulated manufacturing introduces challenges in sterility, cell sourcing, and process validation. Bioprinted constructs intended for implantation must comply with good manufacturing practice standards, which necessitate closed-system printers, traceable bioink formulations, and rigorous batch testing for viability, microbiological safety, and mechanical integrity (Groll et al., 2018).

Variability in bioink composition, crosslinking kinetics, and cell behavior continues to limit standardization. In addition, large-scale manufacturing requires automated systems capable of producing consistent constructs under Good Manufacturing Practice conditions.

The development of closed-system bioprinters, standardized bioink formulations, and validated quality control protocols will be essential to ensure reliable clinical translation (Matai et al., 2020; Pourchet et al., 2017).

## Interdisciplinary collaboration and clinical integration

The successful adoption of bioprinted skin will depend on both technological advances and the development of multidisciplinary clinical ecosystems. Surgeons, dermatologists, biomedical engineers, and material scientists must collaborate from the design phase to ensure that printed constructs meet surgical handling requirements and integrate seamlessly with host tissue. Early clinical protocols should focus on indications where bioprinting offers clear advantages over conventional methods, such as extensive burns, donor-site coverage, or high-precision facial reconstruction (Weng et al., 2021).

Integration into the surgical workflow will also require dedicated bioprinting suites or mobile sterile platforms capable of operating in perioperative environments. The development of standardized imaging-to-print pipelines, linking 3D defect scanning, computer-assisted design (CAD), and automated deposition, could further streamline intraoperative use. As training and reproducibility improve, bioprinted grafts may evolve from specialized interventions to routine components of reconstructive and aesthetic practice (Huang et al., 2025).

## An expanding innovation ecosystem

The evolution of bioprinted skin is driven by a broader technological ecosystem that integrates biofabrication, imaging, artificial intelligence, and materials science. High-resolution optical and ultrasound imaging now allow precise mapping of wound topography and skin thickness, providing input for custom print designs. Artificial intelligence algorithms can optimize cell distribution, print trajectories, and crosslink parameters in real time, enhancing both structural fidelity and cell viability. AI-assisted systems may further improve fabrication through predictive modelling of crosslinking kinetics, automated print-path optimization, and dynamic correction of deposition fidelity during the printing process. Meanwhile, the development of smart and adaptive bioinks, responsive to temperature, pH, or mechanical cues, extends the functional versatility of printed constructs, enabling dynamic responses once implanted (Haykal, 2025).

This convergence blurs traditional disciplinary boundaries: materials science provides the scaffold, AI ensures precision, and clinical dermatology defines therapeutic goals. As these domains integrate, the concept of skin regeneration is evolving from static replacement to adaptive biological design, a living interface between technology and physiology.

## Ethical and regulatory dimensions

The transition of bioprinted skin from research to routine clinical practice raises both regulatory and ethical considerations. These constructs are based on cell banks derived from donor tissues. This approach enables reproducibility and scalability but requires robust ethical oversight. Informed consent from cell donors must clearly specify potential downstream uses, including genetic modification, disease modeling, and integration into biofabricated tissues. Transparent data governance and traceability are essential to ensure donor rights and responsible material use, particularly when digital libraries or “skin avatars” are developed from these standardized cell sources.

From a regulatory perspective, cellular bioprinted constructs are typically classified as Advanced Therapy Medicinal Products in Europe or biologic products under FDA regulation. These categories demand compliance with Good Manufacturing Practice, encompassing donor screening, cell characterization, sterility assurance, and potency validation. Harmonized global standards will be critical to ensure both safety and ethical integrity as bioprinted skin transitions toward clinical implementation (Bhar et al., 2024).

## Future perspectives and outlook

Within the next decade, the clinical translation of bioprinted skin is likely to expand from specialized burn units to mainstream reconstructive and aesthetic surgery. Point-of-care bioprinters may allow surgeons to print custom biological patches intraoperatively, reducing the need for secondary grafting and improving cosmetic outcomes (Jovic et al., 2020).

At the same time, the global shift toward non-animal testing frameworks will drive further investment in standardized, validated skin models, establishing bioprinted skin as both a scientific and ethical benchmark. The convergence of biotechnology, AI, and personalized surgery will ultimately transform skin regeneration from a restorative act into a regenerative, data-driven discipline (Bhar et al., 2024; Budharaju et al., 2025).

## Conclusion

Bioprinted skin exemplifies the convergence of engineering precision and biological realism that defines next-generation regenerative medicine. By overcoming the limitations of conventional grafts and animal testing, it introduces a transformative framework for ethical, reproducible, and personalized skin restoration. Its clinical impact extends far beyond wound coverage, encompassing disease modeling, drug discovery, and tailored therapeutic innovation.

As scalability, regulatory alignment, and interdisciplinary collaboration mature, bioprinted skin is poised to transition from laboratory innovation to a standard component of reconstructive and aesthetic surgery. The future of bioprinted skin will depend on interdisciplinary collaboration, regulatory alignment, and the development of scalable manufacturing strategies, ultimately redefining standards in dermatology and reconstructive surgery.

## Data Availability

The original contributions presented in the study are included in the article/supplementary material, further inquiries can be directed to the corresponding author.
